# Problem characterization for visual analytics in MOOC learner's support monitoring: A case of Malaysian MOOC

**DOI:** 10.1016/j.heliyon.2020.e05733

**Published:** 2020-12-28

**Authors:** Mohammad Fadhli Asli, Muzaffar Hamzah, Ag Asri Ag Ibrahim, Enna Ayub

**Affiliations:** a Faculty of Computing and Informatics, Universiti Malaysia Sabah, Malaysia; b Centre for Future Learning, Taylor's University, Malaysia

**Keywords:** Computer science, Information visualization, Visual analytics, Learning analytics, MOOC, Case study

## Abstract

Malaysia and many other developing countries progressively adopting massively open online course (MOOC) in their national higher education approach. We have observed an increasing need for facilitating MOOC monitoring that is associated with the rising adoption of MOOCs. Our observation suggests that recent adoption cases led analyst and instructors to focus on monitoring enrolment and learning activities. Visual analytics in MOOC support education analysts in analyzing MOOC data via interactive visualization. Existing literature on MOOC visualization focuses on enabling visual analysis on MOOC data from forum and course material. We found limited studies that investigate and characterize domain problems or design requirements of visual analytics for MOOC. This paper aims to present the empirical problem characterization and abstraction for visual analytics in MOOC learner's support monitoring. Detailed characterization and abstraction of the domain problem help visualization designer to derive design requirements in generating appropriate visualization solution. We examined the literature and conducted a case study to elicit a problem abstraction based on data, users, and tasks. We interviewed five Malaysian MOOC experts from three higher education institutes using semi-structured questions. Our case study reveals the priority of enabling MOOC analysis on learner's progression and course completion. There is an association between design and analysis priority with the pedagogical type of implemented MOOC and users. The characterized domain problems and requirements offer a design foundation for visual analytics in MOOC monitoring analysis.

## 1 Introduction

In recent years, Massively Open Online Course (MOOC) rises as a trending online education platform for higher education institutes around the world (Yuan and Powell, 2013). As of 2019, 13,500 MOOCs have been created by over 900 universities globally by the end of the year (Shah, 2019). Due to its flexibility and openness, MOOCs have garnered massive enrolment and participation from learners across the globe. Considering the magnitude, MOOCs usage generates a large volume of complex data, and analyzing them can be a daunting task. Therefore, we observed an increasing need for MOOC analytics considering the increases in MOOC adoption in global education delivery. Our main motivation is to analyze and characterize the design requirements for supporting MOOC analysis with visual analytics. Existing literature on MOOC visualization focuses on enabling a visual analysis on MOOC data from forum and course material (Qu and Chen, 2015; Vieira et al., 2018). Literature shows limited studies that investigate and characterize domain problems or design requirements of visual analytics for MOOC.

This paper reports our empirical investigation in characterizing the domain problem of visual analytics for MOOC support analysis. We investigate specific user and system design requirements of visual analytics for facilitating learner support monitoring. We adopted Munzner's nested model for visualization design and validation (Munzner, 2009) for characterizing domain problems of our MOOC case. We selected Munzner's nested model as a characterization guide for its advantageous structure for mapping visualization design. This model structures visualization design into four levels and recommends appropriate validation methods for each level. The structures and recommendations help in mitigating threats to the validity of the proposed visualization design.

In this paper, we specifically focus on the level of domain problem and data characterization and abstraction. We examined literature and conducted a case study to elicit problem abstractions based on Miksch's data, users, and tasks model (Miksch and Aigner, 2014). In our case study, we interviewed five Malaysian MOOC experts from three different higher education institutes using semi-structured questions. The experts' feedback helps us in characterizing MOOC analysis tasks and analysis scenarios in real-world cases. In addition, we also examined their visualization familiarity by exposing them with two different sets of visualization examples. The experts' visualization familiarity results help us to elicit domain user requirements for designing visual analytics for MOOC data. Translating problem abstractions from domain vocabulary to visualization vocabulary helps in identifying visualization solutions to the problem at hand.

Our study specifically contributes empirical problem characterization and abstraction for deriving design requirements of visual analytics for MOOC analysis. The resulting empirical characterization and abstraction focus on facilitating visual analysis on MOOC data, particularly in learner support monitoring. Visualization designers can leverage our problem abstractions as the groundwork of future visualization system designs for MOOC analysis.

This paper is organized as follows: In Section 2, we present related work in problem-oriented visualization research, visual analytics for MOOC, and MOOC case in Malaysia. Section 3 then describes our methodology in characterizing and abstracting domain problem in visual analytics for MOOC learner's support monitoring. In Section 4, we present and discuss the results from the problem characterization and abstraction. We summarize and characterize the domain problem of MOOC support analysis based on data, users, and tasks. Finally, we provide a conclusion and describe our future work.

## 2 Related works

In this section, we firstly introduce the concept and notable examples of problem characterization and abstraction. Next, we focus on related literature on visual analytics and visualization for MOOC. Furthermore, we examine the literature related to MOOC implementation in Malaysia to characterize the case scenario.

### 2.1 Problem characterization and abstraction

Lam et al. (2011) highlighted that literature shows limited work focusing on problem-oriented visualization considering its importance in visual analytics design and implementation. In addition, Sedlmair et al. (2012) articulated the design study methodology that leverages problem characterization and abstraction in designing visual analytics solution. We introduce some notable examples of problem characterization and abstraction in design studies across domains.

Wagner et al. (2014) investigated the design requirements of visual analytics for behavior-based malware pattern analysis. They articulated the design requirements based on problem characterization and abstraction via literature review, focus groups, and expert interviews.

Riveiro et al. (2017) conducted a design study on enabling visual analysis in anomaly detection on massive multidimensional road traffic data. They demonstrated their anomaly detection design framework on European real road traffic data. Furthermore, they interviewed expert analysts from industrial organizations to evaluate the design and usability of the proposed framework.

Kwon et al. (2018) design study investigates the design requirement for visualizing recurrent neural networks on electronic medical records. They developed RetainVis, an interactive visual analytics system for prediction-based exploration of electronic medical records with improved interpretability. They designed and evaluated RetainVis via iterative design process and participation with medical experts and artificial intelligence scientists.

Devkota and Isaacs (2018) collaborated with computer scientists in abstracting design requirements for visualizing control flow graphs that represent the program's execution path. They examine the scientist's analysis tasks and use of visualization via questionnaires, interviews, and a year-long observation. Based on their findings, they design and propose CFGExplorer, a system for integrated interactions with the control flow graphs program structure. They evaluated CFGExplorer via guided sessions and semi-structured interviews with scientists.

Despite all the given examples, there are yet to be specific problem characterization and abstraction reported for visualizing MOOC data.

### 2.2 Visual analytics and visualization for MOOC

**Figure 1:**
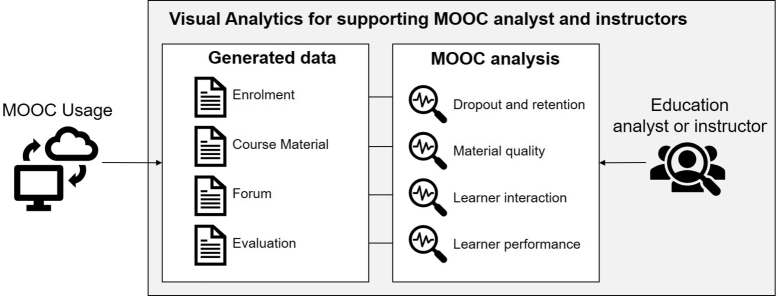
Stages and dynamic of MOOC analysis with visual analytics based on literature review.

Deriving from the following literature survey and review, we can generally describe how visual analytics is utilized in MOOC analysis as illustrated in Fig. 1. We examined the processes of data analytics and visualizations discussed in those surveys and reviews including the type of data, analysis, and target users. MOOC platform generates data from hosted courses' interaction that can be analyzed by analysts and instructors. We categorized the data based on purpose and sources into four categories: enrolment, course material, forum, and evaluation. Each data category can be analyzed separately or combined to support MOOC analysis. We further categorized MOOC analysis based on the focus of observation and data utilization. The four major categories of MOOC analysis are:

1. *Monitoring dropout and retention rates*: We characterize this analysis as the monitoring of learner's enrolment and behavior to anticipate their course completion.
2. *Assessing course material quality*: We refer this analysis as the quality assessment to evaluate the effectiveness of a course module design or material.
3. *Exploring learner interaction*: We characterize this analysis as the investigation of crowd interaction concerning topics of discussion and forum activities.
4. *Evaluating learner performance*: We refer this analysis as the learning performance assessment to ensure fulfillment of pedagogical objectives of the course.

Vieira et al. (2018) investigated existing approaches for visualization designers and researchers in visualizing educational data. Their investigation encompasses visualization method, target audiences, educational analysis purposes, and data sources. They classified the reviewed literature into three dimensions: visualization background, connection with educational theory, and sophistication of visualization.

Qu and Chen (2015) surveyed the current MOOC practices and highlighted research opportunities for visual analytics. They summarized the challenges of MOOC visualization into four categories: data privacy, streaming analysis, personalization, and predicting retention. Our investigation lapses into personalization and predicting retention by focusing on supporting instructors in learner support monitoring.

Bodily and Verbert (2017) recently presented a review on learning analytics and recommender systems focusing on reporting learners' progress and performance. Their findings suggested the need for enabling instructors in analyzing the design processes of these reporting systems. They highlighted that the processes should include needs analyses, visual design analyses, information selection justifications, and student perception surveys.

Literature indicates there remains a gap in problem characterization and abstraction focusing on MOOC learner's support monitoring. Therefore, we perform an investigation to fill this gap and provide a design foundation for future MOOC visualization and visual analytics.

## 3 Methodology

This section describes our methodology for eliciting the problem characterization and abstraction of visual analytics for MOOC learner support monitoring. Firstly, we conducted a literature review to identify existing problem characterizations or design studies on visualizing MOOC data. Next, we conducted expert interviews to understand and characterize domain problems and analysis tasks for Malaysian MOOCs. Besides, we investigated the experts' familiarity with visualization to elicit targeted domain user requirements.

### 3.1 Literature review

**Figure 2:**
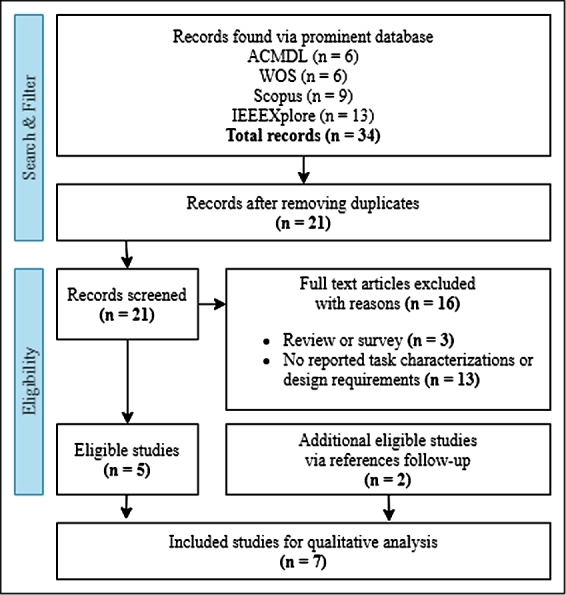
Literature review workflow.

We searched and examined recent visualization articles focusing on design study and MOOC via online scholarly databases like Scopus, IEEE Xplore, ACM Digital Library, and Web of Science. We selected these databases for our literature search to help us retrieve high-quality peer-reviewed articles for review. We searched for articles within 5 years range using these keywords on metadata: *visualization*, *visual analytics*, and *MOOC*. The literature review process is as illustrated in Fig. 2. We further examined these articles and selected relevant studies using the selection criteria described in Table 1. We found 7 notable design studies and applications on MOOC visualization from the selection process and discussed them in Subsection 4.1.

**Table 1:** Inclusion and exclusion criteria for selecting relevant literature for review.

No	Criteria	Justification
1	The study must be an original research paper instead of a review or survey paper.	Review and survey papers do not always contain a sufficient description of design studies or detailed problem characterizations.
2	The study must report design studies for visualizing MOOC data.	To investigate existing domain problem and task characterizations and specific design requirements for visualizing MOOC data.
3	The study must be written in English	English is the common language among the authors who carry out this study.

### 3.2 Expert interview

To help us characterize domain analysis tasks and user requirements, we recruited several MOOC experts from the education field in Malaysia. Firstly, we identified and listed 10 leading MOOC experts and pioneers in Malaysia as candidates for our interview. We contacted and briefed our study to each candidate individually via email and phone call. At the end, we managed to get 5 domain experts that voluntarily agreed to participate. Before the expert interview, we developed an interview protocol for semi-structured interview sessions as follows.

**Interview question and material**: Firstly, we focus on understanding the MOOC scenarios and analysis workflow by asking the experts these questions:

• What is the current state of MOOC implementation in Malaysia?
• What is your interest during assessing information on MOOC data?
• Are there any analytics tool available for the expert?
• Are there any educational theories or pedagogies driving your analysis tasks?
• What is the workflow of your MOOC analysis?

Subsequently, we exposed each domain expert with two sets of visualizations as listed in Table 2, Table 3. Understanding user familiarity with the visualizations helps us in characterizing the specific user design requirements. We further categorized the visualizations in Set 2 into groups of visualization functions based on Abela's chart categorization (Abela, 2008). We displayed the visualizations on paper to each domain experts in similar order and ask the following questions:

• Is this visualization familiar to you and have you used it before?
• Can you understand the information presented by this visualization?

**Table 2:** A set of basic visualization we expose to the experts during the interview.

Set 1: Basic visualization	
1. Pie chart	7. Bubble chart
2. Bar chart	8. Radial chart
3. Column chart	9. Scatterplot
4. Line graph	10. Comparison chart
5. Area chart	11. Stacked bar chart
6. Doughnut chart	12. Visual gauge

**Table 3:** A set of advanced visualization categorized based on visualization function.

Set 2: Advanced visualization				
Function	Comparison (Total=19)	Distribution (Total=12)	Relationship (Total=14)	Composition (Total=18)
Visualization	**Table:** Area-line chart Radar chart Venn diagram Bar chart Line graph Stacked area chart Stacked line-area graph Disparate bar chart 6 view comparative charts Bulls-eye chart Tree bar chart Radial bar chart 3 view comparative charts Data table Variable width column chart 2 view line graph Stacked line-bar chart Stacked radar chart Column chart	**Table:** Timeline 3D line chart Stem-and-leaf plot Timeline plot Dot plots Line histogram Scatterplot Sankey diagram Correlation scatterplot 2 view timelines Trendline scatterplot 3D line graph	**Table:** Area-line chart Venn diagram Process workflow Concentric circles Radial diagram Stacked area chart Bubble chart Topic bubble Stacked line-area graph Word relation flow Sankey diagram Correlation scatter plot Tree map Exploded word relation	**Table:** Heatmap Waterfall chart Pie chart Triangular chart Concentric circles Geo map Timeline Process and grid Exploded pie chart Pinned world map Fishbone diagram Bulls-eye chart Area heatmap Tree map Tag cloud Ternary plot Ring pie chart Heat and area map Arrow chart and map

**Participants**: We recruited 5 education experts in MOOC to participate in the interview sessions as described in Table 4. All the interviewed experts have worked as MOOC instructors in their courses at three different Malaysian higher education institutes.

**Table 4:** Information of the interviewed experts.

Expert	Organization	Institute	Age	Experience	Gender	Education
A	Public University	HEI 1	50-59	7 years	male	PhD
B	Public University	HEI 1	30-39	5 years	male	PhD
C	Private University	HEI 2	40-49	5 years	female	PhD
D	Private University	HEI 2	30-39	5 years	male	MSc
E	Public University	HEI 3	40-49	5 years	female	PhD

**Procedure**: We scheduled an approximately one-hour individual interview with each expert at their office. We documented the interview using audio recording and notes. The results of the interview sessions were then combined and qualitatively analyzed as presented in the next section.

## 4 Results and discussion

In this section, we present and discuss the results from the literature review and case study expert interview. Subsequently, we characterized the problem abstractions for visual analytics in learner's supporting monitoring based on data, user, and tasks.

### 4.1 Literature review

In addition to surveys presented in Section 2, we examined and summarized seven notable studies focusing on MOOC visualization. Moreover, we analyzed Abela's thought-starter chart (Abela, 2008) for shaping our user requirements categorization. Furthermore, we examined and characterized the MOOC case scenario in Malaysia.

#### 4.1.1 Visualization and visual analytics design for MOOC

The emergence of MOOC big data stipulates analytics researchers to develop methods for leveraging it in education analysis. Dernoncourt et al. (2013) presented MoocViz, an open-access large scale analytics that enables analysts in analyzing MOOC data. MoocViz enables the community of education analysts to share and compare MOOC analysis findings. They demonstrated MoocViz's cross-platform capabilities via a joint analysis of two courses from different platforms with great results.

Most MOOC platforms record user interactions log with course materials that allow analysts to analyze learning behaviors. Chen et al. (2015) investigated the design challenges for visualizing multivariate data of MOOC learner's interaction behaviors with course videos. They introduced the system PeakVizor to helps MOOC analysts in determining complex learning patterns in MOOC video interactions. PeakVizor enables MOOC analysts in analyzing clickstream activities in course videos to characterize learner's behavior with learning material. For problem characterization and design requirements identification, they interviewed three MOOC experts to identify analytics tasks and user background. Furthermore, they evaluated PeakVizor's usefulness and effectiveness via case studies and interviews with domain experts. Shi et al. (2015) also developed a visual analytics system namely VisMOOC for analyzing learning behaviors using MOOC video clickstream data. Adopting a user-centered design process, they collaborated with MOOC instructors to characterize analysis tasks and design requirements for VisMOOC. Subsequently, they evaluated and discussed VisMOOC's usefulness via case studies involving the instructors, with new findings on learning behaviors.

Sequential information on learning activities helps MOOC analysts characterize learning behaviors via establishing correlations between learning sequences and performance. Chen et al. (2018) recently introduced ViSeq, a visual analytics system that facilitates information exploration on learning sequences in MOOC. ViSeq helps in mitigating sequential information loss and visualizing learner categorical learning sequence and causality of learning behaviors. They collaborated with several MOOC instructors in characterizing domain problem and task analysis of ViSeq via interviews. Subsequently, they conducted case studies and expert interviews to evaluate the usefulness and effectiveness of ViSeq. Schwab et al. (2016) investigated the existing visualization with regards to visualizing information hierarchies in educational course contents. They presented Booc.io, a visualization system that allows linear and non-linear presentation and navigation of educational concepts material. They derived the design goals of Booc.io using problem abstraction via user studies and expert interviews.

Education analysts examine MOOC online discussion forums to understand learners' activities and opinions on the courses. Wong and Zhang's design study (Wong and Zhang, 2018) investigated the design requirements for their MessageLense tool that facilitates multifaceted analysis of MOOC forums. They developed MessageLens for supporting instructors in analyzing forum from three facets: discussion topic, learner attitude, and learner communication. They conducted a case study on MessageLens's usage using real-world MOOC forum data to demonstrate its design capabilities. Furthermore, they interviewed an experienced MOOC instructor in a preliminary evaluation of the benefits and limitations of MessageLens. Fu et al. (2016) conducted a design study for developing a visual analytics system that enables effective temporal patterns analysis in MOOC forums. They designed the iForum system for visualizing three aspects of MOOC forums in different scales: posts, users, and threads. They collaborated with three domain experts in an iterative manner to characterize design problems and requirements for iForum. Furthermore, they evaluated iForum's effectiveness and usefulness via a case study involving domain experts and real MOOC forum data.

In general, literature shows that existing design studies focus on visualizing learning behaviors in video viewing, module visits, and forum activities. Despite using user-centered approaches, these studies did not deliberately discuss analysis tasks and design requirements in real-world MOOC scenarios. In addition, we also examined Abela's thought-starter chart (Abela, 2008) to help us in understanding the targeted user requirement. We then map our visualization selection material based on Abela's categorization as shown in Table 5. We further discuss the association of our interview participant's visualization familiarity with this categorization in the following section 4.2.2.

**Table 5:**
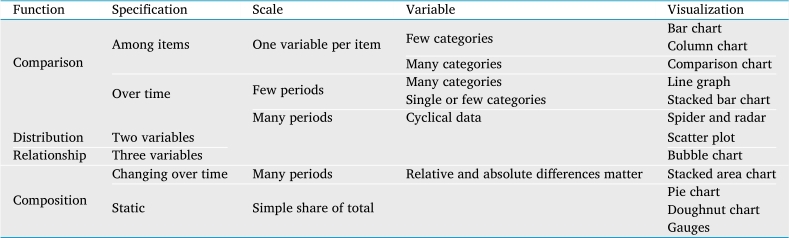
Mapping and categorization of our visualization material based on Abela's thought-starter chart.

#### 4.1.2 MOOC in Malaysia

Albeit many studies focusing on visual analytics for educational data, there remains feasibility or usability challenges in contextual cases. Contextual differences change how visualization designer formulate visual analytics design encompassing analysis tasks, user backgrounds, and accessible data. We discuss the background of MOOC in Malaysia to construct the contextual case for our problem characterization.

Ally et al. (2019) discussed the impact of MOOCs and online learning pedagogies in international context. They characterized the chronology of MOOC development in Malaysia that started in 2013. They discussed the existing online learning approaches to identify key challenges in global online education. Furthermore, they analyzed the Malaysia MOOC Initiative, the first governmental MOOC project in the world. Their findings offer insights for MOOC pioneering efforts encompassing development strategies, learning design and integrative teaching.

Ayub et al. (2018) investigated existing course designs to conceptualize a learning design framework for massive virtual online learners. They described the recent development of Malaysian MOOC as exploratory and focusing on MOOC's acceptance, perception, and effectiveness. We infer that this development is currently necessary for complementing Malaysian higher education delivery. Subsequently, we anticipate that highest analysis priority for Malaysian MOOC is focusing on learner's progression and completion.

Sari et al. (2020) recently investigated the Indonesian and Malaysian instructors perception and strategy towards MOOC instructional design challenges. Their findings reveal four primary design challenges: collaboration encouragement, learner engagement, course material development, and time constraint. Using sequential mixed method, they examine the courses, and then conducted a survey and voluntary interview among 46 instructors. They also noted that hybrid or blended MOOC approach was used in delivering half of the courses.

We observed the current state of Malaysian MOOC including practices and challenges to characterize the contextual case scenario. This scenario leads to unique contextual design requirement and challenges in implementing visual analytics for MOOC support analysis. Therefore, we further investigate for possible contextual design requirements and challenges by carrying out a case study with domain experts.

### 4.2 Expert interview

We conducted semi-structured interviews with 5 domain experts to derive design requirements of future visual analytics for MOOC support analysis. The procedure of the interviews is previously described in Section 3.

#### 4.2.1 MOOC analysis case scenario

**What is the current state of MOOC implementation in Malaysia?** The participants described that the current Malaysian MOOC is focusing on improving teaching and learning course design. They reiterated that MOOC were introduced and reinforced by Malaysian government to supplement national higher education delivery method. Expert A remarked that *“Our MOOC was approached differently; it was a top-down project.”*. An advisory council namely Malaysian e-Learning Council for Public Universities (MEIPTA) was established to develop policies and guidelines of implementation structure for designing Malaysian MOOCs. The council categorizes Malaysian MOOCs into three types: *general* (offered by all Malaysian university), *niche* (university's expert area), and *lifelong learning* (professional development and skill enhancement).

The participants reported that most Malaysian MOOC implemented similar pedagogy as blended learning. Expert B described that *“We are more towards blended learning. Although the learning content is 100% there, but the practice of it is just to help out with whatever face-to-face learning, in general, they are still blended course.”*. Furthermore, Expert D added *“The approach commonly used in most Malaysian MOOCs is turning conventional courses into blended learning. That is why the introduced definition of our MOOC is only massively online course, and then adapted for blended learning and focusing on internal students.”*

**What is their interest during assessing information on MOOC data?** The participants articulated that their current interests mostly focusing on analyzing course enrolment and learner's progression. They oversee information on learner's demographic and monitoring learner's module completion over the course period. Expert C remarked that *“We have to monitor how the students are doing throughout the course. My role as teacher or what you call as instructor, we need to look out for students that may encounter difficulties and provide support. At the end, the module completion percentage in each week is what matters.”*. In addition, Expert E shared *“In my previous MOOC, I learn that my course was participated by students in the other parts of the world. I realized that the course we created not only have benefits our students, but the others outside. Our course has some level of outreach, which is great. The one that participated in my course was a teacher in India. She was having difficulty in getting learning material and resources about the topic, so she enrolled in our course. Eventually, she also recommends our course to her students.”*.

They reported that they need to ensure learner's course completion per requirement set in key performance index by their institution. Expert D explained *“We created our course with regards to blended learning, so it still constitutes a small percentage of credit hour for the program. We have to ensure that all the students completed this part”*. Periodically, they examine discussion threads linked to course material to understand and support learner's comprehension. Expert A stated *“To me, I create some courses, I want to share my knowledge with educators worldwide, so I occasionally visit the course and ask how was their learning.”*.

**Are there any analytics tool available for them?** For report generation and overview assessment, the participants reported that they mainly use the OpenLearning's analytics dashboard. Expert E elaborated *“Since Malaysian MOOC mainly hosted and centralized in OpenLearning platform, most instructors are using the default analytics in there. Although there are some cases, where we export our data and do the analysis outside of OpenLearning.”*.

The participant group HEI1 reported that they also used Gephi and NodeXL for conducting social network analysis. Expert D remarked *“I have experience in using the OpenLearning dashboard, but our work here is focusing on learning analytics. Data can be accessed by our institution, we just asked the data center to provide, then we do our own analysis. Right now, I am using tools for making network graph like Gephi and NodeXL.”*.

Each participant is proficient in extracting and exporting data from OpenLearning, however, they rarely use other visualization tools. In addition, some participants have experience in using Moodle for designing online courses, but exceptionally focuses on analyzing data.

**Are there any educational theories or pedagogies driving their analysis tasks?** Participant group HE2 reported that they are focusing on aligning their course design with the learning objectives. As instructional designer, they design and develop course materials, tutorials, and evaluation according to course's objectives. Expert B explained that *“If you ask me specifically because I am the designer and developer, I am actually looking for the interaction level. I want to look at which activity has low participation. At which activity that students seem to have delayed responding.”*.

They examine learner's interactions with the designed course materials and structures to evaluate the effectiveness of course design. Expert B added *“We want to have a quality MOOC, but what actually are the components that make up for that? So, let us say ‘engagement’, what kind of engagement that the students want? So this is, what they call it as ‘learning design’, but at the end of the day, the output would be something to measure, let us say a lecturer, they come with contents, we need like a system to measure, whether whatever they prepared have actually reached the quality standard.”*.

**What is the workflow of their MOOC analysis?** Each participant developed and hosted their courses in the OpenLearning platform under their respective institutions. They start their analysis by observing overview information on course enrolment and identify learning module progresses. Expert C explained *“When you go the dashboard in OpenLearning, I will first check how many students in the course. And then I will check the overall progress of each student for that week, you know like each week have its own module or chapter. We are in this week, and the students should have progressed up to this chapter.”*.

Next, they analyze the student's learning activities and progression to ensure that students are progressing within session schedule. They identify students with limited progression and investigate the student's learning activities to deduce the causality of limited progression. Expert D remarked that *“Based on the overall list, we can see how many students are falling behind their progress. We look into their activity to understand why like, have they completed this tutorial, did they post something in the discussion, have they received the appropriate response or support.”*. Moreover, Expert E explained that *“Educators are mostly concerned on the causes of events in the course. Is the designed tutorial being too hard for the students? Is the given material sufficient to help the student scores the quiz? We don't want the assessment we set out to be out of current students' comprehension.”*. In addition, they examine and respond to student's thread and comments in the forum to administer learning support.

#### 4.2.2 Visualization familiarity

**Is this visualization familiar to you and have you used it before?** To characterize expected user visual knowledge, we associate the participant's visualization selection with our previous categorization in Table 5. Table 6 displays the participant's selection results on their familiarity with the exposed basic visualization. Basic visualization selection result exhibits user's visual perception challenges in understanding visualization that represents *many data categories* and *temporal perspectives*. In addition, we calculated the selection count percentages to produce participant's visualization familiarity profiles as shown in Table 7. Referring back to categorizations in Table 6, the selection consists of 19 comparisons, 12 distributions, 14 relationships, and 18 compositions. We acknowledged participant E's background as data scientist with visualization expertise so we can focus on other participants. Based on the visualization profiles, we can associate the relationship between the expert's background and their selection of familiar visualization. The participants have used visualization for comparison and composition, but few understands the visualization for relationship and distribution. Expert D explained *“We commonly use regular graph like these visuals in simple visualizations. I don't think I ever used many stacking charts or multiple graphs. I know it is usable, but we need training and knowledge to read it properly.”*

**Table 6:** Domain expert's familiarity towards basic visualization.

Visualization	Participant				
	A	B	C	D	E
Bar chart	x	x	x	x	x
Column chart	x	x	x	x	x
Comparison chart		x		x	x
Line graph	x	x	x	x	x
Stacked bar chart	x	x	x	x	x
Radar chart	x				x
Scatterplot	x	x			x
Bubble chart	x	x			x
Stacked area chart		x	x	x	x
Pie chart	x	x	x	x	x
Doughnut chart		x	x	x	x
Visual gauge			x	x	x

**Table 7:** Domain expert's familiarity percentage towards advanced visualization.

Advanced visualization by function	Domain expert's familiarity (%)				
	A	B	C	D	E
Comparison (total of 19)	42	53	21	16	100
Distribution (total of 12)	25	42	8	0	100
Relationship (total of 14)	21	71	0	0	100
Composition (total of 18)	22	56	11	6	100

The participants also expressed that they preferred user-friendly interfaces and visualizations to be embedded in the design. Expert C remarked *“I prefer the simple visualization you show here instead of that one.”* Expert D also stated *“It would be good if the tool you plan to design is somehow connected with the current system we are using, like when we use the system, there will be an analysis mode. Although it is difficult since we are using third-party host. Perhaps via external link to your tool, but yes, connected with the system.”*

These results evidently indicate that our targeted domain user will largely be comprised of instructors with novice visualization experience. We will encounter several challenges in facilitating visual analysis for novice visualization domain users as highlighted by (Grammel et al., 2010). They have identified and characterized three major challenges in supporting the data exploration process for novice users: data selection, visual mapping, and interpretation. Novice visualization users often rely on heuristics and familiarity with visualization types when attempting to read information from visualizations. Therefore, we infer that leveraging users' familiarity in the design iteration of visual analytics solution allows for easier user adaptation.

**Can you understand the information presented by this visualization?** The participants reported that they commonly understand the information represented using basic visualization. Expert E explained the reasoning *“Unlike data scientist or education analyst, educators do not look at data as we do. They need motivation to come up with inquiries for exploring their data. If your target user is for broad audience of educators, it is wise to use some form of visual that they often use in their regular work.”*

Some participants expressed difficulties to use some of the advanced visualization due to unfamiliarity and limited visualization literacy. Expert B remarked *“I am only familiar with most of the simple one, and only several from the advanced one. I only encounter like this one, fishbone diagram, I saw that before as a framework, I think it was the Blue Ocean Strategy. Most of the time, I only saw or use most of these simple visualizations.”*

They prefer a simple visualization that just clearly represent the information directly relevant to their key performance indicator (KPI)-associated analysis. Expert C mentioned *“I just need to know specific thing about my course, and the fastest way to retrieve the information, so I can answer any inquiries by the faculty.”* Expert A highlighted *“I think if the dashboard is easy to use and the system is brilliant, perhaps. I want to point out that sometimes the educators don't know what question or inquiry to ask when using the system. Data kept accumulated throughout the years, but educators also yet to know how to best utilize it.”*

### 4.3 Data-users-tasks analysis

We abstracted the domain problem and analysis tasks then summarized it using Miksch and Aigner's data-users-tasks design model (Miksch and Aigner, 2014). Their model structures the problem characterization and abstraction into three visualization design aspects:

• Data: What data that the users work with?
• Users: Who are the expected users of the visual analytics solution?
• Tasks: What are the analysis tasks and interest of the users?

**Data**: Malaysian MOOC's instructor mainly host and conduct their course via institutional clustering on the OpenLearning platform. Currently, they use provided analytic dashboard with limited visual analysis features for MOOC monitoring and data analysis. OpenLearning allows the instructors to access or export their course's data via instructor privileges and institutional access. We found that data attributes recorded in OpenLearning is almost similar as another platform like Coursera and Udemy. The data attributes recorded constitutes information on student administration (enrolments and payments) and engagement (posts and course completion summaries). Instructor can access the records from the analytic dashboard and export them for further analysis. However, the records contain limited information for displaying student's individual activities such as individual module completion or peer interaction. OpenLearning data security and privacy policies requires instructors to access the student's individual activities data via institutional access. The participants reported that Malaysian MOOC's structure is characterized as closed participatory that mainly comprised of internal students and instructors. They remarked the rarity of voluntary peer interaction among students and some participants recognized that instructed interaction is naturally lacking compared to conventional forum discussion. It appears that this finding is consistent with the study by (Hew and Cheung, 2014) that indicates lack of peer interaction in many MOOCs. Referring to the MEIPTA's MOOC categories, they participants articulated that general courses is conducted as synchronous learning. Synchronous courses follow the progression of academic sessions and enrolls averagely 300 to 500 students per session. The participants also reported that niche and lifelong learning courses is conducted as asynchronous learning with flexible participation. They added that these types of courses commonly enroll an average of 300 students and more. From our observation and participant's description, we infer that enabling visual analysis for learning support monitoring can be challenging. For Malaysian case, instructor requires visual analysis features that allows temporal observation on individual scale with ease-of-use visualization.

**Users**: The participants reported that MOOC's function in Malaysian higher education can be different between public and private universities. We infer that this difference leads to different analysis interest priorities, though eventually focuses on providing learning support. The participants described that public universities' approach focuses on providing alternative education delivery method. In addition, they articulated that private universities' approach focuses on utilizing MOOC as pre-enrolment support for prospective students. The participants are proficient in using analytics dashboard provided by OpenLearning but have limited exposure on visual analysis. They described the limitation of existing analytics in enabling in-depth analysis such as individual monitoring and peer interactions. They remarked the need for simple visualization design to facilitate instructors with limited or novice visualization literacy.

**Tasks**: Referring to the literature review results, we learn that existing design studies on MOOC Based on expert interview, we recognized that monitoring *student's progression* throughout the courses as primary analysis task. Malaysian MOOCs instructor analyzes temporal progression of student's activities and module completion. We infer that instructor's interest is driven by the need in sustaining course's completion rate to ensure KPI compliance. Due to the session running alongside the academic semester, most instructors concern on student finishing within the end of semester week. For the secondary task, the participants articulated the need for enabling observation on *student's peer interaction* within the courses. The participants highlighted the importance of allowing this observation due to peer interaction direct association with course's completion rate. The participants remarked that self-initiative data exploration is uncommon among instructors due to perception towards standardized MOOC guidelines.

The instances of our case encompass real-world scenarios for available MOOCs in higher education institutes in Malaysia. Considering Malaysia's recent adoption of MOOC, it is likely that we may find similar instances on other countries that share similar MOOC development.

## 5 Conclusions and future work

This paper has presented the empirical problem characterizations and abstractions for visual analytics in MOOC learner support monitoring. Our study offers insights on the explicit design and domain user's requirements for visualizing MOOC temporal events monitoring. The current findings found in this study expand the existing prior work on design studies for MOOC visualization.

We investigated and formulated the characterization and abstraction via literature review and case study expert interviews. The experts enumerated that Malaysian MOOC enrolls moderate number of learners via centralized platform with different course learning mode. Although the courses are centralized, potential data were limited by data tracking scheme and restricted export access. Based on the interview, we identified two major analysis tasks for Malaysian MOOC: monitoring learners' progression and peer interactions. However, we also learn that the instructors were limited by preconfigured analytics to perform these analysis tasks. Our findings reveal that Malaysian MOOC instructors have different analysis priorities that are institutional-dependent. In general, domain user for this case requires simple visualization features with high learnability due to their unfamiliarity with advanced visualization.

We also encountered several limitations in carrying out this study. This study investigated the visualization background of targeted domain user using generic visualization familiarity survey. Although the identified familiarity is sufficient, the proper visualization literacy level for targeted domain users remain to be identified. Future studies can leverage the assessment method by (Boy et al., 2014) to explicitly identify domain users' visualization literacy levels. Moreover, we did not include dataset schemes in another platform apart from the centralized platform used in many Malaysian MOOC. Other MOOC platforms may have extended data tracking schemes including video viewing behavior or detailed forum behaviors or topics. In future, it is advised that visualization designer considers data accessibility due to most potential MOOC data requires institutional access to the hosting MOOC platforms.

Our problem characterizations and abstractions offer supporting groundwork for future design studies of visual analytics for MOOC cases. The characterizations and abstractions can help visual analytics designers in understanding MOOC analysis tasks and evaluating design alternatives. Finally, adopting user-centered design process helps designers and domain experts to understand the real-world case and analysis scenario. This process allows designers to align visualization design development with domain expert analysis requirement and practices. We will collaborate further with participating experts in designing and evaluating visual analytics for MOOC learner support monitoring.

## Declarations

### Author contribution statement

M. F. Asli: Conceived and designed the experiments; Performed the experiments; Analyzed and interpreted the data; Wrote the paper.

M. Hamzah: Conceived and designed the experiments; Analyzed and interpreted the data; Wrote the paper.

A. A. A. Ibrahim: Conceived and designed the experiments; Wrote the paper.

E. Ayub: Contributed reagents, materials, analysis tools or data.

### Funding statement

This research did not receive any specific grant from funding agencies in the public, commercial, or not-for-profit sectors.

### Data availability statement

Data will be made available on request.

### Declaration of interests statement

The authors declare no conflict of interest.

### Additional information

No additional information is available for this paper.

## CRediT authorship contribution statement

**Mohammad Fadhli Asli:** Conceptualization, Data curation, Investigation, Methodology, Writing – original draft. **Muzaffar Hamzah:** Funding acquisition, Resources, Supervision, Writing – review & editing. **Ag Asri Ag Ibrahim:** Supervision, Writing – review & editing. **Enna Ayub:** Resources, Validation.
